# The eco-ethical contribution of Menico Torchio – a forgotten pioneer of European Bioethics

**DOI:** 10.1186/s13010-023-00145-5

**Published:** 2023-12-20

**Authors:** Iva Rincic, Amir Muzur, Cristina Richie

**Affiliations:** 1 Department of Social Sciences and Medical Humanities, University of Rijeka, Faculty of Medicine; Head, Rijeka, Croatia; 2 Department of Public Health, University of Rijeka, Faculty of Health Studies, Brace Branchetta 20, 51 000 Rijeka, Croatia; 3 Ethics and Philosophy of Technology Section, Delft University of Technology, Delft, Netherlands; 4 University of Edinburgh, Edinburgh, Scotland, UK

**Keywords:** Bioethics, Fritz Jahr, Menico Torchio, Van Rensselaer Potter, Environmental bioethics, Biomedical ethics

## Abstract

**Background:**

In 1926, Fritz Jahr described bio-ethics (German: bio-ethik) as “the assumption of moral obligations not only towards humans, but towards all forms of life.” Jahr summarized his philosophy by declaring, “Respect every living being on principle as an end in itself and treat it, if possible, as such!.” Bioethics was thus originally an ethical system concerned with the “problems of interference with other living beings… and generally everything related to the balance of the ecosystem” according to the 1978 Encyclopedia of Bioethics. This definition was predicated on the work of Fritz Jahr, Menico Torchio, and Van Rensselaer Potter.

**Methods:**

In order to proceed with depthful analysis of the origin and major bioethical flare up, we will use critical analysis of existing literature, followed by a study trip to relevant bioethical localities (collecting photo and other documentations regarding Menico Torchio).

**Results:**

While Jahr and Potter are typically given intellectual credit for developing the field of bioethics, the eco-ethical contributions of Menico Torchio have been forgotten.This article will first trace the origins of “bioethics” – now commonly bifurcated into “biomedical ethics” and “environmental bioethics.” The former was developed by Tom Beauchamp from the Philosophy Department and James Childress of the Religious Studies department at Georgetown University and is based on principlism, with a narrow focus on medical settings. The latter addresses the environmental impact of the medical industry and climate change health hazards. Second, we will present a panorama of Torchio’s significant intellectual contribution to bioethics. Menico Torchio’s concept of bioethics synthesized work of both Jahr and Potter, advocating “the need to expand our ethical obligations and embrace the most developed groups of animals, not only physically but also psychologically.” Third, we will reflect on the lasting legacy of “bioethics” on biomedical and environmental bioethics today. Thematic elements such as interconnectedness of planetary health and human health, dedication to living in harmony with nature, and emphasis on systems and symbiosis remain unchanged from the legacy of Tochio onward.

**Conclusion:**

Our conclusion will underscore the necessity of understanding the connections between planetary, environmental, and human health.

## Background

Codes of professionalism and ethics were initially contained within the domains of the physicians guild and integrated with training. From the beginning of medicine, “ethics” education has been part and parcel of socialization into the profession of medicine. Take, for example, the statements of Asclepius on the ethics of treating someone with an incurable disease and the social benefits and drains of the ill in Plato’s Republic. Plato records that the physician Asclepius “did not think it worthwhile to treat a man incapable of living a normal life since such a one is of no use to himself or to the state” [1]. That is, extending life merely for the sake of respiration and circulation is not the purpose of medicine; here social and medical ethics are one in the locus of the physician in service of the State. “Medical deontology” was the standard form of ethics education in Soviet medical schools [2]. Significant for the history of medical ethics was the development outside of the medical practice.

Theologians were among the first “bioethicists” to address ethical decision-making in medicine as an outgrowth of moral theology [3]. The long history of moral consideration in health care has characterized nearly every society where both religion and medicine were present. In Catholicism, for instance, a rich system for adjudicating the morality of medical dilemmas was produced, tracing back to moral manuals like Heribert Jone’s *Moral Theology* (1946) [4]. Of course, many of the principles employed in bioethics were developed long before Jone, starting with Thomas Aquinas (2008) [5]. Catholic moral principles have received commentary at various times by theologians who used casuistry (a case study methodology) to apply historically accepted principles to contemporary medical and moral dilemmas [6–8].

In United States health care, starting from the mid-1900s, the principle of totality (or integrity) and the distinction between ordinary and extraordinary means were utilized by pioneer theologians like Gerald Kelly [9] as having relevance for amputation in the former case and end of life care, in the latter. The principle of the double effect, cooperation, and proportionalistm, taught by James Gustafson [10, 11] was relevant for terminal sedation, medical abortion, and experimental therapies, respectively. Gustafson’s theological reflection influenced both his Catholic and Protestant students [12–15] who made major contributions to globally-focused health care, end of life and beginning of life issues, and disability studies. The influence of Catholic theology on medical ethics was prominent in other mid-century scholars [16–19]. These developments in medical ethics were unique in the “audience” they were addressing. Medical ethics, here, was offered as guidance clergy, who in turn would guide the laity. These developments in medical ethics were not intended to be normative for secular medical professionals, even though medical professionals could have had a cosmological commitment which aligned with these reflections.

Later the development of biomedical ethics—a discrete discipline where a non-physician could be an expert on moral matters related to medicine—emerged [20]. Daniel Callahan cites Joseph Fletcher’s book *Morals and Medicine* (1954) as “the first truly fresh manifestation of a growing interest in medical ethics in the post-World War II era” [21]. He also notes that contemporary, non-religious biomedical ethics from a non-clinician perspective emerged as a discipline “during the 1960s and 70s in an era of affluence and social utopianism…(and) for medicine, it was a time that combined magnificent theoretical and clinical achievements with uncommonly difficult moral problems” [21]. Outside of medical schools, Centers dedicated to bioethical inquiry, which were comprised of theologians, philosophers, lawyers, policymakers, and doctors—like the Hastings Center—emerged.

Other significant developments that influenced medical ethics outside of the clinical setting include Paul Ramsey’s book, *The Patient as Person *[22], a 1974 conference on bioethics at Haverford College [21], and the Belmont Report [23]. The culmination of these developments are apparent in theories underpinning modern day medical ethics. Particularly, in Western liberal societies where the pursuit of health and longevity is often in tension with other social values, like sustainability, expense, and access, balancing moral boundaries with boundless scientific developments require discernment filtered through ethical theory.

### Methods

In order to proceed with depthful analysis of the origin and major bioethical initiatives we will use critical analysis of existing literature, followed by a study trip to relevant bioethical localities (collecting photo and other documentations regarding Menico Torchio).

## Results

Probably the first on the European continent after Fritz Jahr, the term "bioethics" was used after a 35-year hiatus in June 1973 by the Italian Menico Torchio (1932–2001) [24, 25]. Born in Turin and raised in Eritrea, he gave up living in a Benedictine monastery due to illness (although he was the secular oblate of the abbey in Savona from the age of 17), he turned to the study of natural sciences and graduated from the University of Turin with a thesis in "eco-ethology.^1^" From 1968 to 1982, he ran the Milan Aquarium,^2^ reviving the hydrobiological station and founding the Quaderni della Civica Stazione Idrobiologica di Milano periodical. After a short engagement at the University of Cagliari, he became a professor at the Institute of Animal Ecology and Ethology of the University of Pavia (since 1983 the Department of Animal Biology, and today the Department of Earth and Environmental Sciences). Permanently interested in the history and philosophy of science, and especially in the cultural offerings of the Benedictines [30]^3^. Torchio retired on July 1, 1996 [32] into isolation, not understood even by his few close friends.

In addition to books on his predominant occupation – oceanography, translated into several languages (he was the first to describe a species of fish, Arnoglossus moltonii Torchio: the name is not generally accepted), Torchio published several other articles on bioethics, particularly intrigued by "bioethical intuitions" in the works of Greek early Christian writers [33]. Declaring himself a „Mazzinist “ (in other words, believing in a republic imbued with a strong faith of the people), he was consistently active in public life: when environmentalism began to strengthen in Europe and the United States in the early 1970s, Torchio became secretary of the „G. Gadio “ Group for basic ecology (Gruppo "G. Gadio" per l'ecologia di base; the name is somewhat reminiscent of the idea of A. Næss' "deep ecology"), named after the street where it originated (at the City Hydrobiological Station), founded in May 1971 in Milan, around which university teachers, museologists, and amateurs gathered to promote environmental awareness through conferences, publications and in other ways [34].

In March 1984, he founded the "Ecosystem Analysis Group" at his Department in Pavia [35]. Although Torchio's 1973 article, in the title of which he mentions bioethics ("Man-Nature Relations to Major Eastern Metaphysics, Their Bioethical and Ecological Implications") [27]. is undoubtedly driven by Potter's bioethics—in terms of pointing out the dangers of disturbing natural balance in the biosphere due to the neglect of cultural and ethical factors – it is amazing how similar this article is to Fritz Jahr's key article from 1927: first, Torchio's article was published in the highly read and respected journal *Natura—rivista di scienze naturali *(published since 1909 by Italian Society for Natural Sciences and the City Museum of Natural History in Milan), just as, in Jahr's time, was the *Kosmos – Handweiser für Naturfreunde und Zentralblatt für das Bildungs- und Sammelwesen*, published in 1904–1999. (when the title was changed to *Natur + Kosmos*) by the Stuttgart Association of Friends of Nature; second, the title of Torchio's article ("Man-Nature Relationships…") is similar to the subtitle of Jahr's article („Bio-Ethik: eine Umschau über die ethischen Beziehungen des Menschen zu Tier und Pflanze “); third, Torchio in his article advocates the recipes of Eastern metaphysics as potentially salutary for the ecological sins of Western civilization, and Jahr, as it is well known, in the 1927 article (and only in it) introduces the philosophies of Buddhism, Yoga, and Sankya as examples of correct behavior towards the living world [36]. Torchio analyzes in detail the principle of non-violence – the *ahimsa*, admiring especially Ghandi about whom he will write a special article and give a lecture in 1982, while the article we are talking about, from 1973, will be dedicated to the 25th anniversary of Ghandi's death)^4^; fourth, Torchio mentions in several places in the article the understanding of ethics as a “force that resists egoistic instinct,” and Jahr is known to have devoted an article precisely to the “opposition and unification” of egoistic and altruistic principles [37], finally, as the fifth: in the second of only two mentions of “bioethics” in his article, Torchio cites the term “bioethical imperatives” and in several places cites the wording of the Padma Purana “do not do to others what you do not want to yourself” (in other places, Torchio cites similar variations of the “Golden Rule,” such as that of Confucius or Hillel Sr.) [38].

These similarities should be complemented by Torchio's later approach to Jahr, when, in his 1983 and 1984 works, he advocated "the need to expand our ethical obligations and embrace the most developed groups of animals, not only physically but also psychologically" [39]. Of course, Torchio does not mention Jahr in the 1973 article,^5^ but neither Potter, nor in the text nor in the bibliography. It is not impossible that Torchio knew about Jahr, and it is quite certain that he quickly learned about Potter: it turned out that Torchio had received Potter's book as a gift in 1972 from the director of his institute (C. F. Sacchi) who had returned from America [40]. How close he was to the Potterian idea (which he came up with, just like Potter, from the non-medical, natural science side) also is shown by the coincidence that in February 1971 – a month after the publication (still unknown to Torchio) of Potter's first book on bioethics – he published an article "State of Alarm" in which he argues that man, as a homeothermic being, consumes enormous amounts of energy in relation to heterotherms, and that man thus in itself is, in fact, a "luxury for the ecosystem." Pointing to humanity's age-old practice of disposing of waste by throwing it into rivers and seas – which, by the synergy of demographic, technological-industrial and urban explosions a few decades ago, exceeded the autopurification capacity of water – as well as soil pollution caused by agriculture and industry, Torchio concludes by using the metaphor of mankind as the „cancer of the entire biosphere “ and by posing the question „do intelligent beings exist on Earth at all” [41].

In a public lecture in May 1974, Potterianly entitled "Bioethics – a bridge to survival" (later published as an article of the same title, again in *Natura *[42], Torchio already mentions Potter, but also quotes Aldo Leopold, Albert Schweitzer,^6^ Giorgio Nebbia (b. 1926, chemist, fighter for clean energy and water, Member of the Italian Parliament and Senate), Bernhard Häring (1912–1998, German Catholic theologian-Redemptorist^7^) and other authors, always emphasizing the medieval "preparation" of bioethical ideas [43, 44]. In one paper, Torchio even highlights, as a final message, his contribution to "naturalistic (and ecological) bioethics" (Bioetica naturalistica ed ecologica), at least as dignified as "production bioethics" (Bioetica procreatica), which is "in trend today, perhaps even too great” [45]. Unfortunately, Torchio did not further elaborate on this – as usually – incidental mention of bioethics. That he, in any case, understood the dangers and illogical narrowing down onto biomedicine, according to which the dominant bioethics was heading, is revealed by the conclusion or "comment from a bioethical perspective" of the article on fir-tree cultivation by the Benedictine author Antonio Luigi Fornaini (1755–1838), where Torchio notes: "As far as Bioethics […] is concerned, I am worried about the danger of limiting it, in Italy, to medical schools, and I draw attention […] to its importance for the preservation of the biosphere" (in support of which Torchio cites even Fidel Castro and UNESCO's 1981 „Man and the Biosphere “ Programme. In any case, Torchio's early referring to (Potter's) bioethics (which Torchio did not consider a „fortunate “ term, even if he recognised that he „considered himself a student – even if undeserving – of his 'brother in Bioethics,' Professor Van Rensselaer Potter" [46], quickly fell into oblivion,^8^ suppressed by a major bioethical project to be launched in Italy only a few years later by the Catholic Church.^9^

## Discussion

Of course, a second way of defining bioethics appeared in academia and medicine—biomedical ethics—based on principlism and narrowly focused on medical settings. The so-called Georgetown mantra—respect for patient autonomy, beneficence, non-maleficence, and justice—which was developed by Tom Beauchamp from the Philosophy Department and James Childress of the Religious Studies department at Georgetown University became the standard ethical system for philosophy departments and medical schools. Following from this formalization of biomedical ethics, numerous research centers connected to universities and hospitals arose, focused on the four principles of bioethics to the exclusion of Jahr and Potter’s original concept. Thus, the environmental component to biomedical ethics was forgotten by students, teachers, and practitioners. And while the development of bioethics as an academic discipline gave the appearance that ecology was separate from medicine, environmental bioethics has brought the two together.

## Conclusion

Environmental bioethics, which at once addresses the environmental impact of the medical industry and climate change health hazards, is a dynamic discipline [49]. Simultaneously, thematic elements such as interconnectedness of planetary health and human health, dedication to living in harmony with nature, and emphasis on systems and symbiosis remain unchanged from the legacy of Tochio onward.

Health care emits a significant amount of carbon in many countries [50]. Carbon dioxide emissions do not stay within national borders and contribute to climate change and climate-change related health hazards. When the carbon impact of health care is evaluated, it is primarily at the institutional level—that is, the carbon of hospitals [51].

Many health care organizations including Healthcare Without Harm [52], Practice Greenhealth [53], the Healthier Hospitals Initiatives [54], the Catholic Health Association [55], Catholic Health Association and Practice Greenhealt [56], Catholic Health Association of the United States [57], Catholic Health Initiatives. [58] and UK’s National Health Service [59] and others [60] have recognized the connections between the carbon emissions of external health care and climate change. These, and other, organizations, have implemented initiatives such as recycling and clean energy purchasing.

Moreover, the environmental bioethics movement, tracing to the conceptual groundwork of Fritz Jahr [61], Menico Torchio, and Van Rensselaer Potter [62], the concept of Sustainable Medicine, in the tradition of Daniel Callahan [63] and new work on Green Bioethics [64] argue for healthcare resource reduction from a distinctly conservationist lens. Today, ecological initiatives voice already established ethical concerns, such as public health and highlight new concerns, like distributuve health care justice. The future of sustainable health care will surely vary based on coutnry and interest of medical systems. However, the eco-ethical contributions of those who build the field will be embedded in the activism.

## Data Availability

Not applicable.

## Footnotes

1 Interestingly, it was biologists (Scottish animal geneticist Conrad Hall Waddington, 1905–1975, Ukrainian evolutionist Theodosius Dobzhansky, 1900–1975, etc.) who decisively influenced Potter's formulation of bioethics [26]

2 On the history of the Aquarium, see Torchio (1973), Mojetta (2010) and Polezzo (2006) [27–29]

3 He also assumed that Galileo Galilei was a Benedictine oblate, in any case under the significant influence of the Benedictines. In his study of Galileo, Torchio advocates the coexistence of scientific and religious (intuitive) knowledge in the Benedictines, and perhaps in Galileo himself (Torchio 1982a). Torchio is also interested in the relationship between faith and scientific spirit in other monks, such as the Augustinian abbot Gregor Mendel [31]

4 See also the encomium to Ghandi. In an unpublished manuscript from March 1995, Torchio also declared himself a "Gandhian Catholic" (cattolico ghandiano). (Letter in the personal archives of Edoardo Razzetti.).

5 It remains unknown whether Torchio understood German: older zoological literature is certainly largely in German, but it is also true that, although there are sources in English and French he cites, he does not mention German references.

6 Torchio will also return to Schweitzer in a lecture entitled "The Man-Nature Relationship in the Life and Thoughts of Francis of Assisi," held in January 1982 and later published as article [43]

7 Since 1971 on the Kennedy Institute of Ethics Council in Washington.

8 It is often stated that Torchio's work was "noticed" even in the USSR, which is supported by a summary prepared and published for the Institute for Scientific Information of the USSR Academy of Sciences by Ju. V. Morozov. However, judging by Torchio's correspondence, it seems that Torchio had sent the work to the Academy, which only reacted in the usual way.

9 More on the development of Italian bioethics see in Muzur and Rinčić (2018) and Rinčić et al. (2021). [47, 48]
